# Publishing ethics in the era of paper mills

**DOI:** 10.1242/bio.056556

**Published:** 2020-10-28

**Authors:** Rachel Hackett, Steven Kelly

**Affiliations:** 1 The Company of Biologists, Bidder Building, Station Road, Cambridge CB24 9LF, UK; 2 Department of Plant Sciences, University of Oxford, South Parks Road, Oxford OX1 3RB, UK

In 2013, a journalist working for the journal Science launched a ‘sting’ operation to highlight perceived flaws in the peer-review processes operated by Open Access journals (Bohannon, 2013). Biology Open (BiO) was one of the Open Access journals tested – the submitted ‘nonsense’ paper was rejected without sending for review (Hackett et al., 2013).

As noted at the time, “Despite the policies, checks and balances that have been implemented [on BiO], it can be difficult to identify and block papers that include flawed work or outright fraudulent data.” Back in 2013, it might have appeared that such scientific misconduct was rare. However, much has changed. Resources such as PubPeer and Retraction Watch provide platforms that enable the scientific community to document and catalogue issues of concern with scientific publications, many of which can only be explained by the presence of falsified data. Moreover, the work of Dr Elisabeth Bik and colleagues has begun to expose the extent of fraud in scientific publishing (Bik et al., 2016). The emerging truth from these community efforts is uncomfortable; it appears that misconduct when publishing scientific research runs much deeper than the occasional ‘sting’ operation. Moreover, it appears that fabricated research papers are readily available for purchase by scientists driven by the need to publish more papers to further their careers (see Byrne and Christopher, 2020).

The vast majority of ethical scientific publishers are grappling with this problem. Although there are advanced technological solutions for the detection of plagiarised text (e.g. the iThenticate plagiarism detection software), equivalent solutions for the detection of plagiarised data or data manipulation are less well developed. As exemplified by the efforts of the community of scientists who contribute to PubPeer and Retraction Watch, detection of fraud in scientific data is still best performed by scientists themselves. Thus, many publishers now employ data sleuths to examine manuscripts for evidence of potentially falsified data. There is a pressing need for technological development in this area.

Despite employing scientists to evaluate the data, using plagiarism detection software, and ensuring best publishing practices are followed with respect to peer review, it is difficult for journals to protect themselves from those intent on committing misconduct. BiO, as with many other journals, seems to have fallen victim to this fraud.

The publisher of BiO – The Company of Biologists (a not-for-profit organisation dedicated to supporting and inspiring the biological community) – employs a Publishing Ethics Coordinator, whose role it is to investigate potential ethics issues, before and after publication, as well as older historical cases highlighted on sites such as PubPeer. All accepted articles on BiO are checked for text plagiarism using the iThenticate plagiarism detection software, which checks for plagiarism against cited journal content and 70 billion current and archived web pages. Although no assumptions of wrongdoing are made, any issues that are flagged are escalated to the Publishing Ethics Coordinator and, if necessary, to the journal Editor-in-Chief. COPE – The Committee on Publication Ethics – may also be consulted. Recruitment of independent experts has been undertaken when helpful. Particularly serious cases could lead to rescinding of the acceptance decision and escalation to the authors’ institutes. Post-publication issues could lead to the publication of an Expression of Concern and possible retraction, according to the guidelines recommended by COPE (and outlined in BiO's journal policies).

Figures represent a more significant challenge to detectives working to identify scientific misconduct. Guidelines are provided to BiO authors concerning unacceptable practices. Figures within accepted articles are checked by trained in-house production staff for evidence of image manipulation. Byrne and Christopher (Byrne and Christopher, 2020) have identified two types of fraudulent images: invented images and stock images. Invented images are typically western blots, and might include placing individual bands onto false backgrounds, non-linear adjustments, the splicing of multiple images to suggest they come from the same gel, duplicated bands and lanes, and grouping or consolidation of the data (e.g. removal of lanes) (see Box 1). Stock images are more difficult to detect and show none of the manipulation likely from the production of invented images. They tend only to be spotted by eagle-eyed readers if repeated in multiple submissions or publications. This is beyond the reach of our production team, which is generally able to spot manipulations within and across images within individual articles (for examples, see Box 1). This has prompted the Company to more urgently investigate sophisticated manipulation detection software, which is currently being trialled.

**Box 1. General examples of inappropriate figure manipulation**

Adjustments should be applied to the whole image so no specific feature of the original data, including background, is obscured, eliminated or misrepresented as a consequence. Any alterations, such as non-linear adjustments (e.g. changes to gamma settings), must be disclosed.

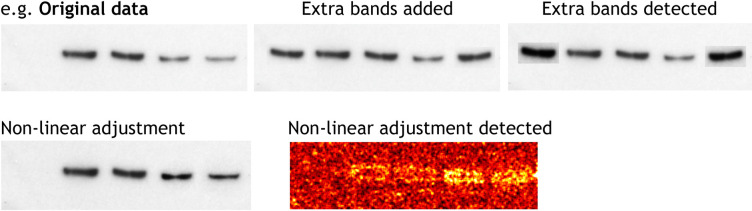

The splicing of multiple images to suggest they come from a single micrograph or gel is not allowed.

Any grouping or consolidation of data (e.g. removal of lanes from gels and blots or cropping of images) must be made apparent (i.e. with dividing lines or white spaces) and should be explicitly indicated in the figure legends.

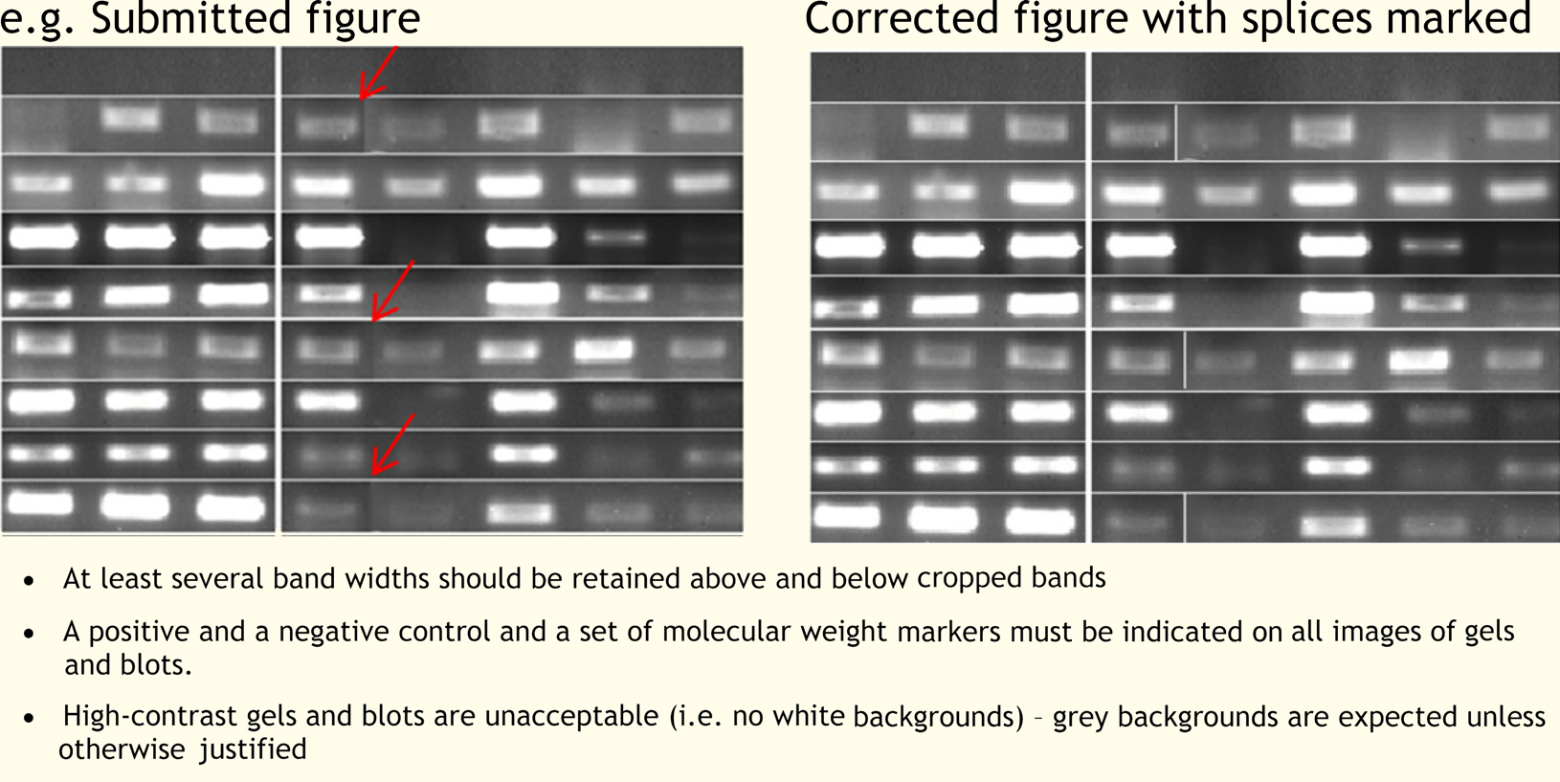

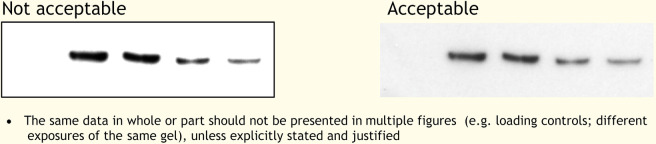

If any inappropriate figure manipulations are detected, again this is escalated to the Publishing Ethics Coordinator in the first instance. They will ask the authors for all the raw data underpinning the results and then conduct a forensic analysis. If problems are detected after acceptance, but before publication, the acceptance might be rescinded. For articles that have already been published, further steps could be taken as outlined above.

For BiO, during 2019, pre-publication issues arose in 9% of accepted articles and four issues were raised post-publication (including historical articles). We published no Publisher's Notes/Expressions of Concern, no Corrections and no Retractions relating to potential ethics cases raised during 2019. In 2020 to date, we have published Expressions of Concern relating to two papers, as a result of concerns rasied by the Editor-in-Chief about the data presented. Our investigations are ongoing.

BiO also strives to adopt best practices with regards to author contribution rules, management of conflicts of interest and reporting of experimental subjects. Peer review is a focus too. To prevent the problem of fake reviews, whereby authors attempt to manipulate the peer review process, reviewers are now required to provide institutional email addresses or ORCIDs (as are corresponding authors at submission and all authors at revision).

BiO publishes peer-reviewed original research in all areas of the biological and biomedical sciences. It is essential that the work addresses a clearly stated, non-trivial, biological hypothesis. It must, in the opinion of the Editor, enhance the literature and be of use to the community. One charge levelled at Open Access journals is that they are more likely to accept submissions regardless of the quality of the research, as that it how they become profitable. Unfortunately, there are numerous so-called predatory journals that do just that. However, the current rejection rate – 67% of articles submitted to BiO are rejected – should absolve BiO of any accusations of predatory practices. BiO and its research-active academic Editors are firmly focused on providing a service to the community by publishing rigorously conducted research. BiO also supports early-career researchers by publishing its hugely popular First Person interviews alongside research articles. BiO has created career development opportunities through our Meeting Reviews and Future Leader Reviews programme. BiO and The Company of Biologists will together continue to strive to ensure that the profits from the hard work of scientists inspire future scientific discovery and help develop the next generation of researchers.
